# Spatio-temporal monitoring of health facility-level malaria trends in Zambia and adaptive scaling for operational intervention

**DOI:** 10.1038/s43856-022-00144-1

**Published:** 2022-07-01

**Authors:** Jailos Lubinda, Yaxin Bi, Ubydul Haque, Mukuma Lubinda, Busiku Hamainza, Adrian J. Moore

**Affiliations:** 1 School of Geography and Environmental Sciences, Ulster University, Coleraine, UK; 2 School of Nursing, Ulster University, Belfast, UK; 3 School of Computing, Engineering and Intelligent Systems, Ulster University, Derry, UK; 4 Telethon Kids Institute, Malaria Atlas Project, Nedlands, WA Australia; 5 School of Computing, Ulster University, Belfast, UK; 6 Department of Biostatistics and Epidemiology, University of North Texas Health Science Centre, Fort Worth, TX USA; 7 Department of Geography, University of Florida, Gainesville, FL USA; 8 Emerging Pathogens Institute, University of Florida, Gainesville, FL USA; 9 Malaria Institute at Macha/Macha Research Trust, Choma, Zambia; 10 Ministry of Health, National Malaria Elimination Centre, Lusaka, Zambia

**Keywords:** Epidemiology, Malaria

## Abstract

**Background:**

The spatial and temporal variability inherent in malaria transmission within countries implies that targeted interventions for malaria control in high-burden settings and subnational elimination are a practical necessity. Identifying the spatio-temporal incidence, risk, and trends at different administrative geographies within malaria-endemic countries and monitoring them in near real-time as change occurs is crucial for developing and introducing cost-effective, subnational control and elimination intervention strategies.

**Methods:**

This study developed intelligent data analytics incorporating Bayesian trend and spatio-temporal Integrated Laplace Approximation models to analyse high-burden over 32 million reported malaria cases from 1743 health facilities in Zambia between 2009 and 2015.

**Results:**

The results show that at least 5.4 million people live in catchment areas with increasing trends of malaria, covering over 47% of all health facilities, while 5.7 million people live in areas with a declining trend (95% CI), covering 27% of health facilities. A two-scale spatio-temporal trend comparison identified significant differences between health facilities and higher-level districts, and the pattern observed in the southeastern region of Zambia provides the first evidence of the impact of recently implemented localised interventions.

**Conclusions:**

The results support our recommendation for an adaptive scaling approach when implementing national malaria monitoring, control and elimination strategies and a particular need for stratified subnational approaches targeting high-burden regions with increasing disease trends. Strong clusters along borders with highly endemic countries in the north and south of Zambia underscore the need for coordinated cross-border malaria initiatives and strategies.

## Plain language summary

Malaria is an infectious disease that is widespread in many African countries. Malaria transmission within a country can vary between regions, so tailored interventions for malaria control and elimination targeted to different regions are necessary. To achieve this, it is important to measure and monitor the frequency of malaria infections, its risk, and trends at different geographic administrative scales. This study analysed over 32 million reported malaria cases from 1743 health facilities in Zambia between 2009 and 2015. The results showed an increasing national trend in malaria risk and malaria infection frequency and identified differences between health facility and district trends. These findings support a flexible approach when implementing and expanding national malaria monitoring, control and elimination strategies, especially in areas bordering countries where malaria is widespread, cross-border movement is common, and cross-border initiatives could be beneficial.

## Abstract (web summary)

Lubinda et al. analyse over 32 million health-facility reported malaria cases in Zambia (2009–15) to examine spatially-structured temporal trends. They observe overall increasing trends in risk and rates and highlight the potential benefits of using an adaptive scaling approach in national malaria strategies, and a need for cross-border initiatives.

## Introduction

Malaria remains one of the leading causes of death in children and pregnant women in sub-Saharan Africa^1^. With global progress in reducing incidence rates now levelling off, malaria is generally rising, especially in some high-burden African countries. As a result, most of Sub-Saharan Africa, including Zambia, is pursuing malaria policies and strategies to reduce case incidence. Reducing the population proportion living in high-risk areas and pursuing local pre-elimination and subsequent elimination status remains a priority goal for countries^2^.

As local and subnational malaria reduction depends on individual exposure to infectious mosquito bites, local mosquito density, and infectivity, tailored strategies are essential for various local contexts to best use the limited resources available. The need for strong health surveillance systems to inform appropriate intervention programmes, fine-scale monitoring of patterns, and adaptation of strategies as malaria transmission declines have become critical.

This is especially true for most Sub-Saharan countries like Zambia, which are pursuing multiple approaches targeted at local malaria control in some settings and pre-elimination or elimination in others. Targeted interventions, particularly in elimination settings, aim to interrupt local transmission as it becomes increasingly concentrated in small areas that are often very hard and costly to reach^3^. Understanding the spatio-temporal scale dynamics of prevailing malaria epidemiology is imperative to facilitate and successfully target and control those remaining residual reservoirs and infection hotspots^4,5^.

For Zambia, focal targeting of low-transmission settings, alongside accelerated control of malaria in high transmission settings, could help quicken the reduction of malaria burden and hasten progress towards the malaria elimination goal. Besides, the current funding pressure dictates that available, often limited resources are more effectively directed at targeting those areas and populations where the most impact can be achieved^6–8^. With considerable spatial and temporal variability generally inherent in malaria transmission within endemic countries^4,9^, targeted interventions across the transmission spectrum that operate at much finer resolutions than before are a practical necessity for understanding, monitoring, and ensuring effective control^8,10–14^.

Like many sub-Saharan African countries, Zambia’s local malaria policy decisions are primarily based on routinely collected data from its national Health Management Information Systems (HMIS), the District Health Information System (DHIS2). While such sources continue to experience data quality issues such as incompleteness and accuracy and often lack the equivalent scale of treatment-seeking information necessary for making adjustments, they are still the best available sources for strategic and operational planning. Over time, continued improvements in the quality of this data make it highly valuable in everyday decision-making as it requires less and less adjustments for analytical purposes.

As Zambia is one of those countries concurrently pursuing intensified control strategies along with pre-elimination and elimination strategies, approaches targeted at the health facility level have gained momentum, particularly following the WHO’s recommendation to use a malaria continuum measure rather than a uniformly applied strategy^15,16^. This entails identifying and targeting the hardest-to-reach malaria hotspots in the all-important elimination phase or high-burden areas in the intensified control phase. Such an approach supports the design, monitoring, and adaption of appropriate intervention programmes as malaria transmission declines further.

This study uses data at the lowest administrative (health facility) level to investigate the spatially structured temporal trends that characterise fine-scale malaria burden in Zambia. We compare spatio-temporal trends within the health facility and district-level models to identify key differences that could provide policymakers and disease surveillance experts with relevant operational-level evidence to help them develop and plan more cost-effective, targeted, multi-scale intervention strategies. Our findings will help improve the geospatial identification and stratification of areas with high or increasing burdens and improve targeting efficiency in allocating health service resources for malaria intervention planning.

## Methods

### Malaria datasets used in the study

Authorisation for the study in Zambia was obtained from the Zambia National Health Research Authority, and overall permission to use routine malaria data was granted by the Ministry of Health. Ethical approval to perform the study was granted by the Zambian ethical review board—ERES Converge IRB (Ref: 2017-Sept-011). The requirement for individual consent was also waived by the ethical review board, given that all the data were already aggregated to administrative units and contained no individual identifiers.

We obtained aggregated monthly reported malaria case data for 2531 operational health facilities from the Zambian HMIS/DHIS2 from 2009 to 2015. Of the total, 612 (24.2%) are new health facilities that typically reported zero malaria cases throughout the study period and are therefore excluded. The absence of these health facilities was verified against a detailed government health facility census of 2012^17^.

Another 76 facilities, randomly spread across the country, are excluded from the analysis because of incomplete malaria data and/or lack of baseline population information as a denominator in calculating incidence rates and standardised risk ratios (here referred to as risk). Of those health facilities excluded, eight had complete malaria data from 2009 to 2015, ten only had data for 2014 and 2015, and the remaining 58 had data for 2015 only and data collection only started in the second half of the year. The reported malaria case data for 76 health facilities accounted for 0.8% of all recorded cases over the study period. Finally, 100 referral hospitals where severe malaria cases are admitted and treated are separated from the dataset. This separation helped avoid double counting of malaria cases already captured at the lower-level health facilities.

**Fig. 1:**
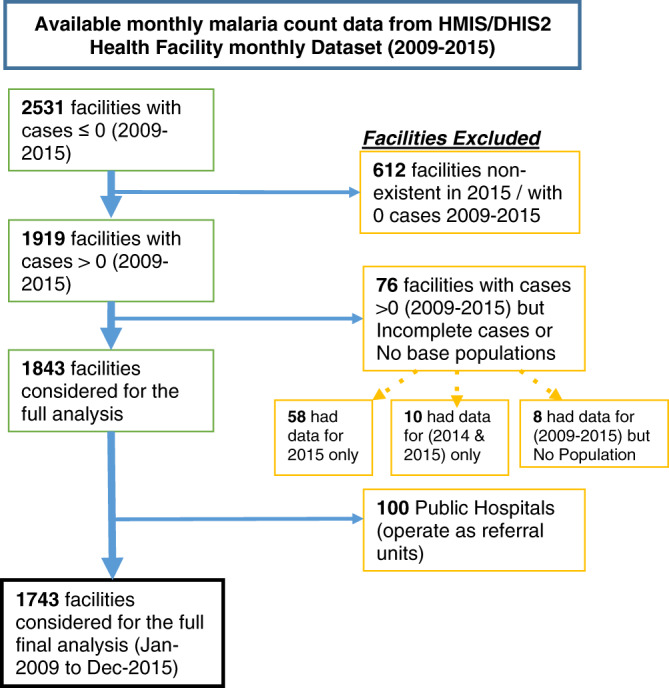
Summary schema of final health facility-level analysis. A workflow of the process of cleaning the dataset and the exclusion/inclusion criteria applied to arrive at the final dataset analysed.

For the same reason, all hospital-affiliated health centres (HAHCs) in this analysis are considered and included as separate health centres/units offering services similar to those of normal health facilities not affiliated with any hospital. All private hospitals are included because they also provide services offered by lower-level public health facilities. The remaining 1743 facilities comprised the complete dataset analysed (see Fig. 1*and* Supplementary methods). Our final working dataset includes 146,000 monthly health facility reports (from 1743 facilities), capturing over 32 million cases in seven years (January 2009 – December 2015). It is worth noting that the exclusion of the eight health facilities could be a potential source of unknown bias in the data by slightly underestimating any effects in the results presented.

The numerator consisted of positive malaria cases diagnosed and confirmed using RDTs, microscopy slides, and adjusted clinically diagnosed cases. Although data are reported in available age categories of <1, 1–4, and ≥5 years, the study population included all ages at risk of malaria, from infants to adults. The facility service base populations, used as denominators in calculating malaria rates, are derived in consultation with the programme and are based on recalculated census health facility service catchment data or reported facility headcounts. Priority is given to headcounts over census estimates.

### Spatial and temporal models implemented

We implemented a Bayesian trend model and a spatio-temporal Integrated Laplace Approximation (INLA) model to analyse spatio-temporal trends over the 7-year study period^18,19^. We used the Integrated Laplace Approximation (R-INLA) package approximation strategy^18^, as implemented in the R programme version 3.5^20^. Given the extensive number of observations in the dataset, we chose the *simplified Laplace approximation*, which is relatively less computationally intensive than the full Laplace but still retains similar accuracy^18^.

### Spatio-temporal model description of integrated nested Laplace approximations

The study area is divided into health facility catchment areas. We used the observed number of malaria cases (*O*_*it*_) in a health facility area *i* at time point *t*, the total population at risk of malaria is *N*_*i*,_ and the expected number of cases (*E*_*it*_) for a health facility area *i* at time point *t* to estimate the malaria incidence ratio. A simplified equation of the standardised incidence ratio (SIR) calculation is shown by the equation:

1 $${SIR}={O}_{{it}}/{E}_{{it}}$$

The SIR is calculated to estimate the risk of malaria at the health facility. In this study, risk and incidence denote the estimated polygon risk or rate, and the population denominator is the population at risk within that facility unit polygon or facility point (as opposed to the true health-centre catchment area).

In our analysis, we implemented a re-parameterised Besag, York, and Mollié model^21^ (BYM) model, which is called BYM2^22^, with the spatial random effect parameter, presented as

2 $$\xi =\frac{1}{\sqrt{{\tau}_{\xi }}}\left(\sqrt{{\lambda }_{\xi }{u}_{* }}+\sqrt{1-{\lambda }_{\xi V}}\right),$$

Where u_*_ denotes the scaled intrinsic Conditional Autoregressive (iCAR) model with a generalised variance = 1, and *V* are unstructured random effects. To avoid identifiability problems, a sum-to-zero constraint ${\sum }_{i=1}^{n}\,{\xi }_{i}=0$ must be imposed. The expression of a weighted average covariance of matrices to the structured and unstructured spatial components comprises the variance of the overall random effect^23^.

The prior spatial distribution implemented through the BYM2 model fits the spatio-temporal model with a random temporal effect and is implemented with a temporal structured random walk of second-order (RW2). For a temporary structured random effect $(\gamma 1,\ldots ,\gamma T)$, we assume a prior distribution to be $\gamma \sim N({0}{,}{[{\tau}_{\gamma }{R}_{t}]}^{-})$ where *T*_*γ*_ is the precision parameter, and *R*_*t*_ is the structure matrix of RW2 (*T x T*) taking after an iCAR prior to the spatially structured variability. We assumed a Gaussian exchangeable prior distribution to model unstructured heterogeneity for the space-time interaction random effect ${\delta} =(\delta _{11},\ldots ,{\delta }_{1T},\ldots ,{\delta }_{n1},\ldots ,{\delta }_{{nT}})$ as $\gamma \sim N\left(0,{\left[{\tau}_{\delta }{R}_{\delta }\right]}^{-}\right)$, where, *T*_*δ*_ is the precision parameter, while *R*_*δ*_ represents the corresponding spatial and temporal structure matrix of *nT x nT* for the full interaction effects. For more details about the BYM2 model implemented, refer to refs. ^22,24,25^.

The incorporated iCAR prior distribution is represented by

3 $$\xi \sim N\left({0}{,}{\left[{\tau}_{\xi }{R}_{s}\right]}^{-}\right),$$

Where *T*_*ξ*_ denotes a precision parameter, and *R*_*s*_ represents the n × n spatial neighbourhood matrix known by a diagonal of elements equal to the sum of neighbours of each area. The non-diagonal elements (*R*_*s*_)_*ij*_ is equal to −1 whenever *i* and *j* are neighbouring areas; otherwise, (*R*_*s*_)_*ij*_ = 0, and *N* is the number of neighbours with the joint distribution. Two areas are defined as neighbours if they share a common border or edge. Here the neighbourhood structure is defined by the matrix of nonzero elements, given by extracting the structure directly from the shapefile.

We also leveraged computation power from the shiny *SSTCDapp*^26^ to run several comparative model fitting performance tests to determine and select the model with the best fit, using comparisons of deviance information criterion (DIC). We applied spatio-temporal models with prior distribution for the spatial random effect of the BYM2. The BYM2 model contains both an intrinsic conditional autoregressive (ICAR) component and an ordinary random effects component for spatial autocorrelation and non-spatial heterogeneity^27^. The model allows all parameters to have clear reading and a straightforward selection of hyperpriors^27^. The models are implemented with a random walk of order 2 (RW2) prior distribution for the random temporal effect and an unstructured temporal random effect. We added a space-time interaction of type (ii) random effect term to account for spatial and temporal autocorrelation.

We computed a spatial neighbourhood matrix from an ESRI shapefile within which two health facilities are considered neighbours if they share a common border or edge. We used the queen criterion to compute the adjacency binary matrix *W* of spatial polygons, defined as *w_ij* = 1 where area “*i*” and “*j*” share a common border or edge, and 0 otherwise. We then fitted health facility Voronoi polygons to generate a unique geographic area with an associated year ID for the temporal variable with RW2. The RW2 assumes that variables take periodic random steps away from previous values, using independently and identically distributed (iid) size steps. While other studies may apply probability measures to density metrics to define populations within catchments of a public health facility^28^, catchment areas in this study had populations assigned from a census estimate or headcount and are only used for mapping, visualisation of trends, and cluster analysis purposes. The Voronoi polygons represent arbitrary exclusive facility delineations that do not reflect the true catchment extent and do not contribute to the definition of catchment populations or incidence estimates.

### Bayesian trends model in a Markov Chain Monte Carlo environment

Finally, we implemented a separate Bayesian Poisson trend mixed model with change point parameters to detect health facility-specific malaria trends over the study period. This is built on methods and models previously implemented for the district-level trend values to compare the health facility and district-level scales^29^. The models are implemented in a Markov Chain Monte Carlo (MCMC) environment, with a burn-in of 10,000, a sample of 110,000, and four parallel chains, with a thinning of the degree of 10. We used Gelman’s trace plots and visual diagnostics to determine the convergence of the models^30,31^. The model structure and equation of the temporal model are denoted by:

4 $${Y}_{kt} \sim p({\mu }_{kt}),\,\,k=1,\ldots ,K, t=1, \ldots ,N, \\ g({\mu }_{kt})={O}_{kt}+{{X}^{T}}_{kt} \, \beta +\phi _k + \, \mathop{\sum}\limits_{s=1}^{s}{\omega}_{ks} \, {f}_{s}(t|\gamma_{s})$$

Where malaria trends *f*_*s*_*(t*|*γS)* estimated in the study are represented by (a) Constant trend—β_1_; (b) Linear increasing trend—β_1_ + γ_1t_, with γ_1_ > 0; and (c) Linear decreasing trend—β_1_ + γ_2t_, with γ_2_ < 0. A more detailed description of this model is given elsewhere in^32,33^. We also used ESRI’s ArcGIS 10.6 for the optimised hotspot analysis.

### Reporting summary

Further information on research design is available in the Nature Research Reporting Summary linked to this article.

## Results

### Spatio-temporal patterns of health facility-level malaria rates and risk

**Fig. 2:**
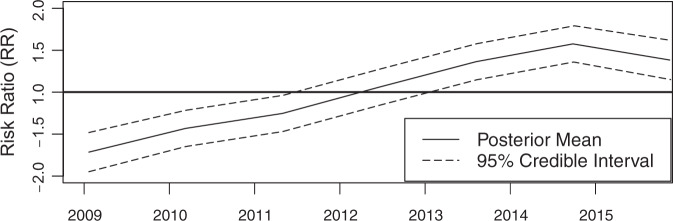
Posterior mean temporal trend of the risk ratio from 2009 to 2015. The solid line shows the posterior mean risk ratio (RR) over the study period. The dashed lines show 95% credible intervals (CI) of RR.

**Fig. 3:**
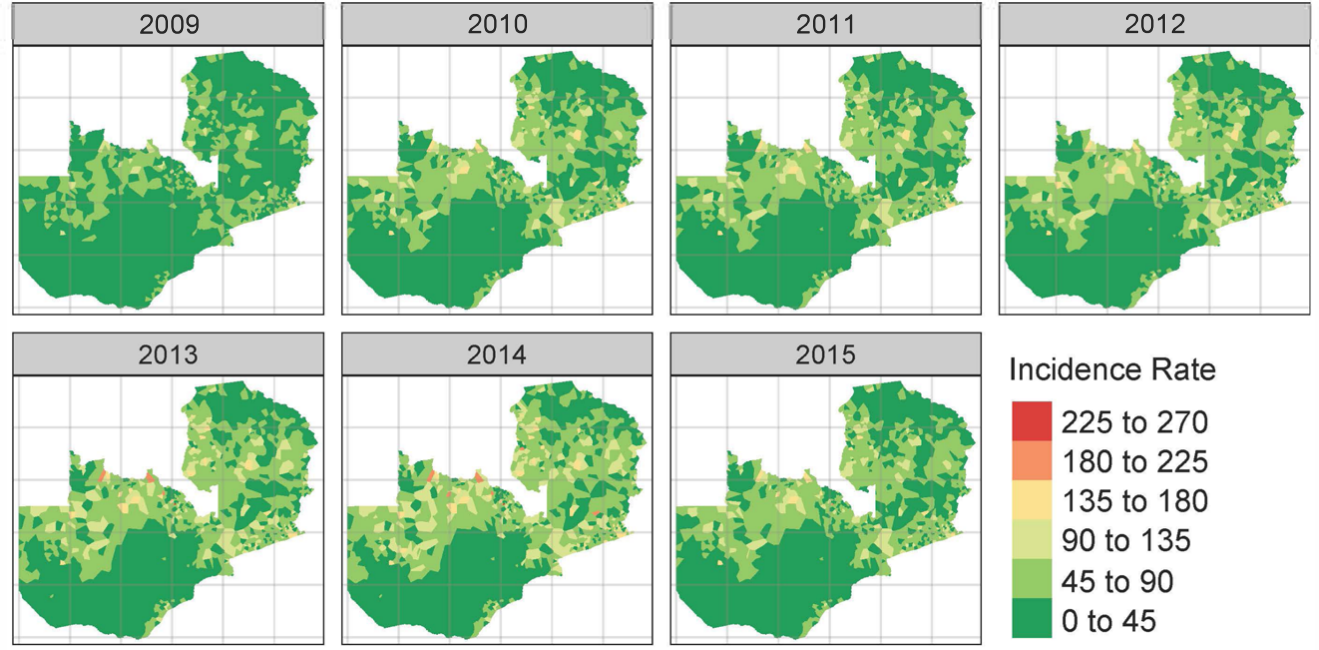
Annual posterior median estimates of malaria incidence per 1000 population, 2009–2015. Deeper green shading indicates lower posterior median estimates while light green - yellowish-brown and red show progressively higher median incidence posterior estimates.

**Fig. 4:**
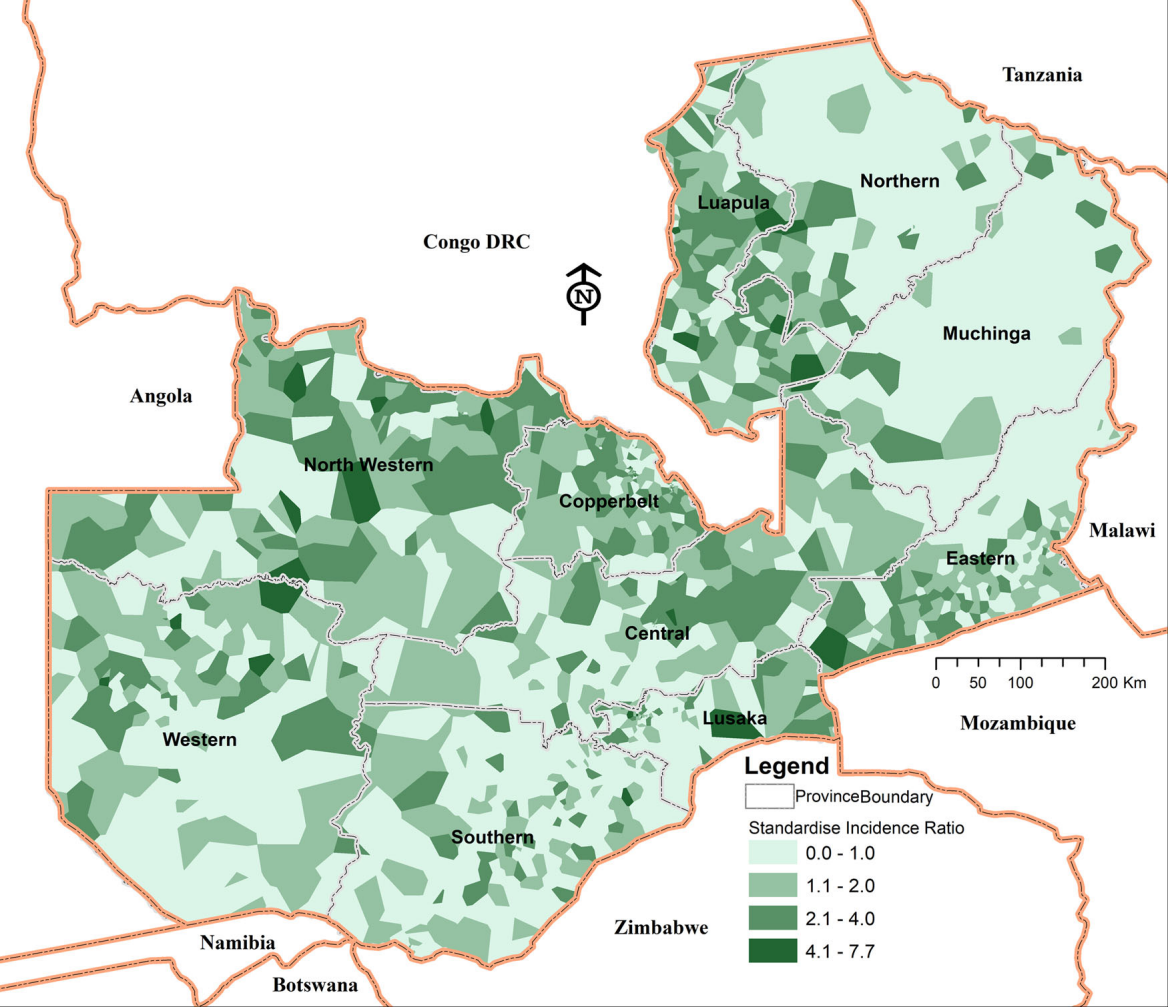
Standardised malaria mean risk ratio, 2009–2015. The colour indicates the mean standardised incidence ratio. Lighter shades indicate lower risk ratio values, while darker shades indicate a higher value.

There is an increase in the national average trend in malaria risk ratio (Fig. 2), and incidence (Supplementary Fig. 1*)* at the health facility level between 2009 and 2014, with only a slight decline in 2015). Figure 3 shows the spatio-temporal trends presented through posterior median estimates of malaria incidence. The temporal pattern shows a southwards shift and an evident shrinking of the number of areas of low malaria. Meanwhile, the mean spatial distribution pattern (see also Supplementary Fig. 2) mimics that of malaria risk presented in Fig. 4, where large areas of low malaria risk ratios (RR) are observed in the country’s southern parts with progressively increasing incidence rates as one moves northwards.

#### High-risk patterns in border areas—the potential of cross-border malaria initiatives

High malaria risk is observed in the northeastern parts of Zambia, mostly around the Luapula province, with the high-risk pattern spatially extending in a southeastern direction towards the Mozambique border. This high-risk area follows the national boundary with the Democratic Republic of Congo (DRC), covering the North-Western, Luapula, Central and Copperbelt provinces. Other noticeable high-risk health facilities areas are further southeast along the Mozambique border.

**Fig. 5:**
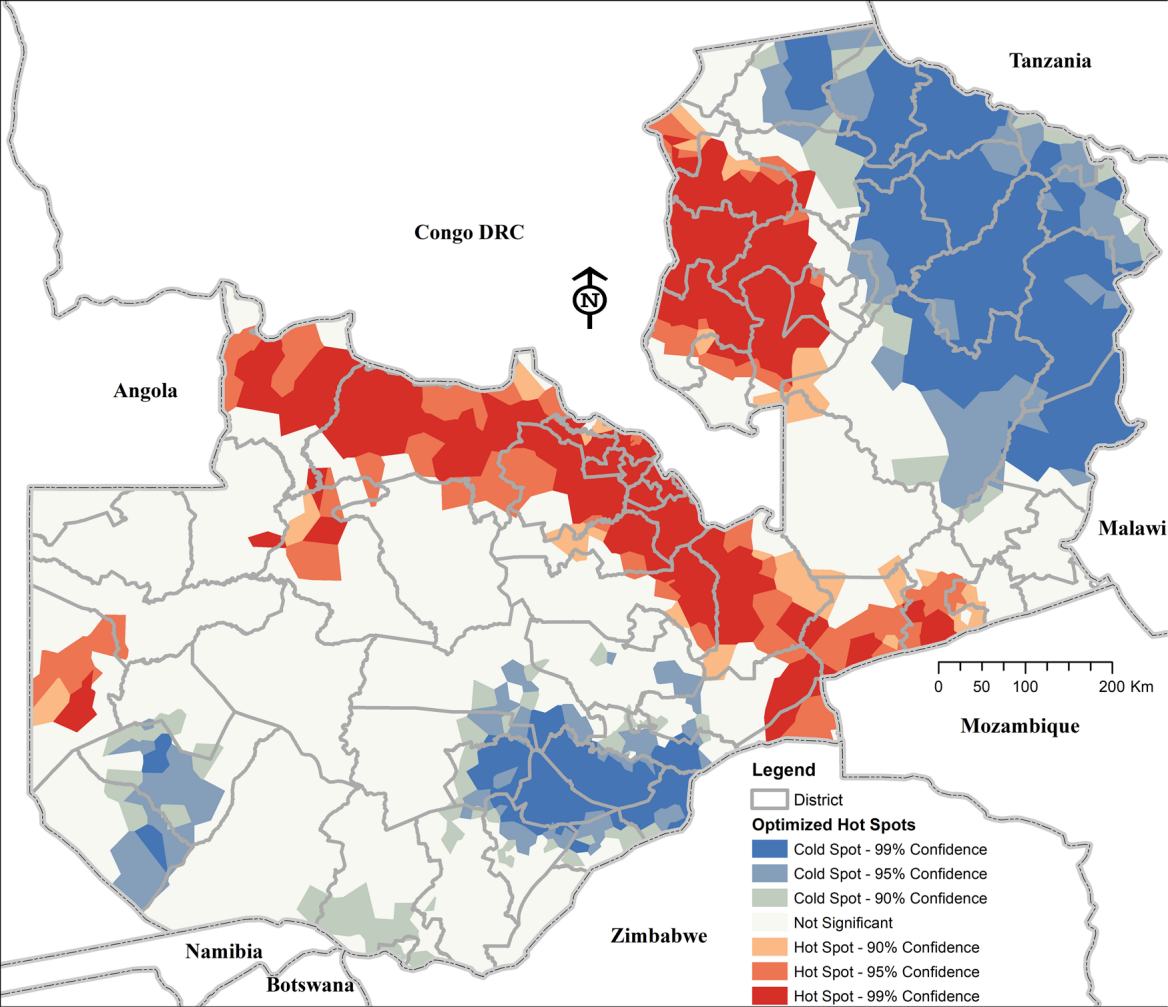
Statistically significant malaria high-risk (hotspot) clusters and low-risk (cold spot) clusters. Colours indicate different types of clusters: brown colour—hotspot clusters of health facilities with higher risk surrounded by areas of relatively lower risk; light to blue—cold spot clusters of lower risk than the surrounding areas. Shades of each colour represent the corresponding significance of results (i.e. white—non-significant); progressively darker depending on how small their *p* values are.

Using optimised hotspot detection methods, we further find consistent statistically significant malaria high-risk cluster hotspots comprising 578 (33%) health facilities and relatively low-risk cold spot clusters comprising 484 (27%) health facilities (Fig. 5). Based on cluster size, intensity, and statistical significance, the two most notable consistent hotspot clusters follow Zambia–DRC and Zambia-Mozambique borders, while some distinct areas of relatively lower-risk (cold spots) compared to their surrounding areas are in the northeast and southeast of the country. Within both the hot and cold spot clusters, minimal variability is detected through outlier detection tests (see Supplementary Fig. 3).

#### Regional and facility-level spatial, temporal and spatio-temporal malaria incidence trends

Detailed results from the model indicate that at least 5.7 million people from 27% of health facilities (469/1743) live in areas with a significant average (linear) declining trend, and 5.4 million people from 47% (826/1743) of health facilities live in areas with a significant average (linear) increasing trend. Only 4.4 million people from 26% of health facilities (448/1743) live in areas with no significant trend change (see Supplementary Table 1 for significance levels). Health facilities with a decreasing trend are more likely to be located in urban areas with much larger catchment populations than those with increasing trends that tend to be more rural with smaller populations (hence the differences in health facility percentages and population numbers).

The results from our R-INLA model using posterior means of incidence rates *(*Supplementary Fig. 4*)* and posterior exceedance probabilities show strong spatial, temporal, and spatio-temporal patterns in the data. Fifty cases per 1000 population is the threshold set by the Zambian national malaria elimination programme to denote areas targeted for intensified control intervention measures, whereby areas with annual cases <50 are considered appropriate for malaria elimination. Further details of the posterior marginal distribution summary statistics using variance and precision scales are given in Supplementary Tables 2,3, respectively.

**Fig. 6:**
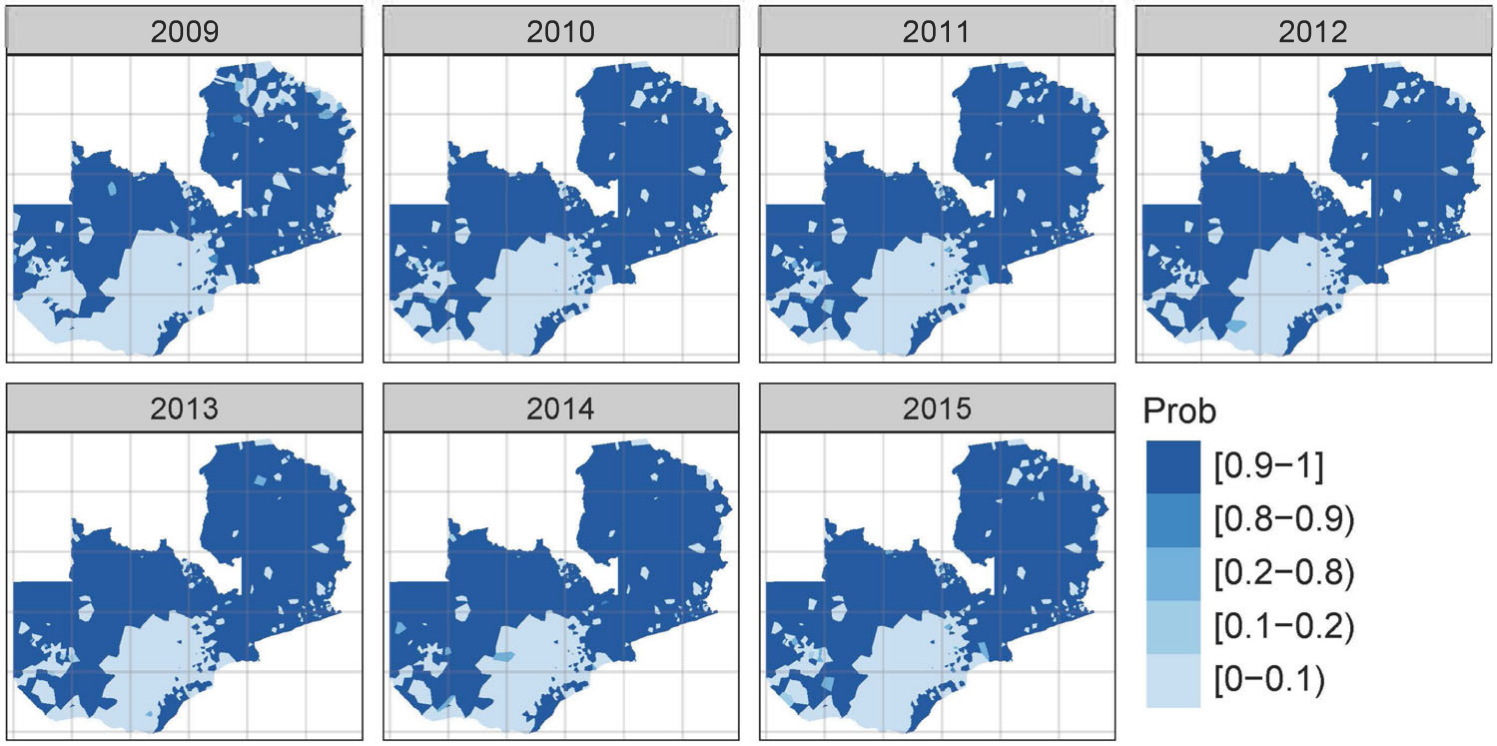
Posterior exceedance probabilities (greater than a threshold of 50 cases per 1000 population). The posterior exceedance probability threshold is 50 based on stratification used by the country at the time. Light shades indicate a lower posterior exceedance probability or a lower likelihood that the area has a higher incidence than expected, while darker shades indicate a higher than expected probability.

The posterior exceedance probabilities are the likelihood that areas have a higher incidence than expected, as shown in Fig. 6. Higher exceedance probabilities occur predominantly in the northern regions, similar to the pattern of mean incidence rates presented in Fig. 3. Of note, however, is the unique and continuous statistically significant (95% credible intervals) annual increase in the number of health facilities reporting higher than expected rates over the seven years with an equally distinct north-south spatial drift. The decrease in spatial heterogeneity observed might imply that while the 50/1000 cut-off makes the starting point for identifying what areas have low malaria suitable for pre-elimination interventions, it may not accurately represent any variations currently experienced in areas of higher or increasing malaria.

**Fig. 7:**
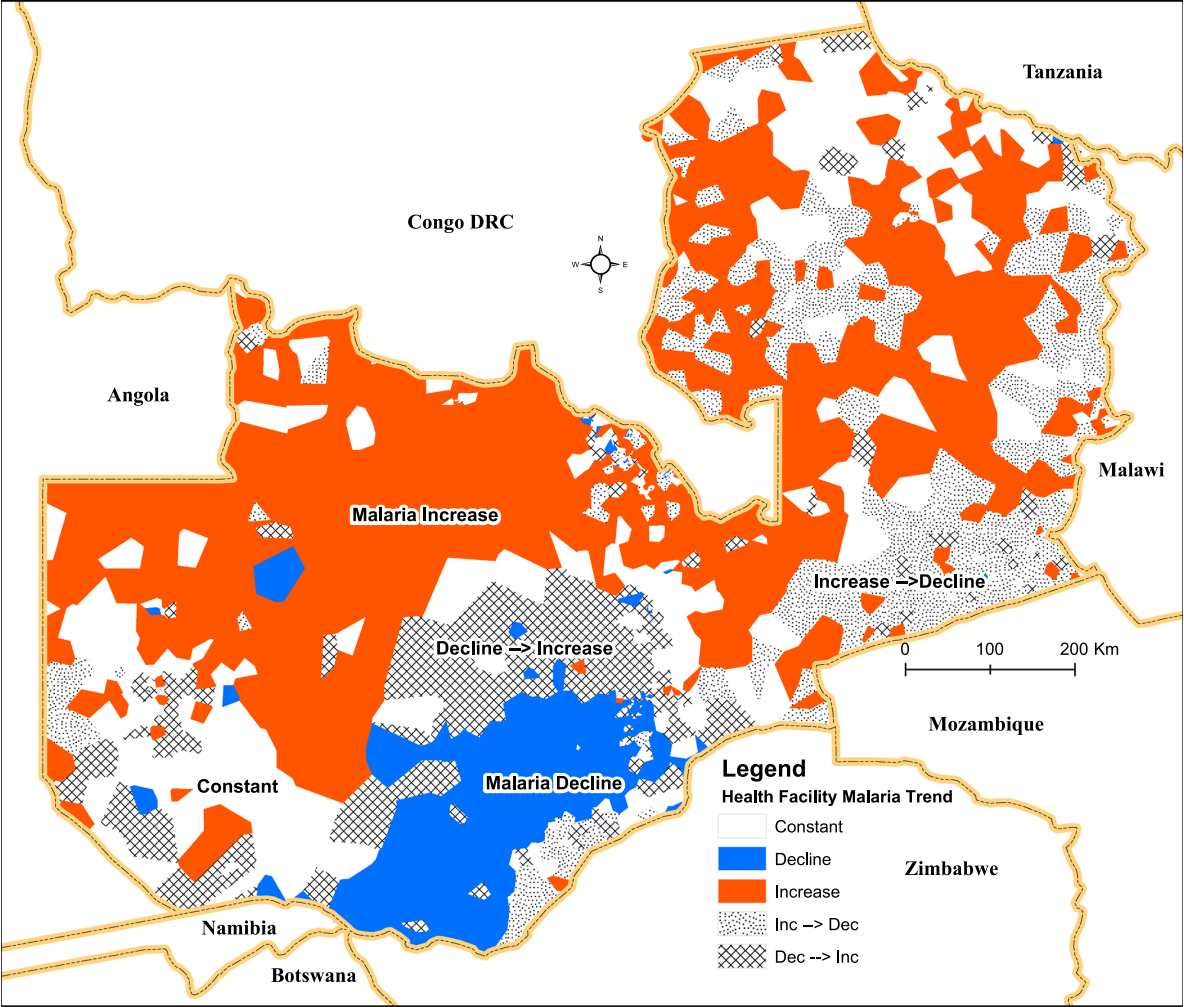
Health facility malaria incidence trends between 2009–2015 (95% credible interval). Colours indicate different temporal trends of areas: Orange shades—areas with continuous increasing malaria trend; blue shades—clusters of areas with declining trends; hatches—areas with a trend changing from declining to increasing after a change point; interlaced black dots—areas with trends changing from increasing to declining; and plain white—areas where no statistically significant linear trend is present.

The modelling of incidence trends indicates a strong spatial context to the stratification of areas moving from increasing to decreasing trends and vice versa (Fig. 7). Generally, southern Zambia exhibits a declining trend. These areas are adjacent to other parts of south-western Zambia with a mix of no significant trend (no change), partially declining, or increasing trends. In northern Zambia, many areas with no significant trend change in malaria tend to be contiguous with areas of increasing trends. At the same time, there are few areas with declining trends in this region. Further modelling of the seven-year trends using the year 2011 as the baseline change point produced a detailed five-class stratification*,* namely: continuous yearly Increase, continuous yearly Decrease, Constant/not change, change from Increase to Decrease, and change from Decrease to Increase.

The continuous yearly increasing trend category accounted for the highest proportion of health facilities (31%), followed by areas from an increase to a decrease (19%). Those facilities with a decreasing or constant/no change trend accounted for 18%, while the proportion of areas moving from a decrease to an increase accounted for 12%. There is a clear continuum of spatial transitioning of regions from being areas of declining malaria to areas of increasing malaria. For instance, in the southern province, the concentration of areas with declining malaria (blue) is encircled by areas whose trend is moving from declining to increasing (hatched) (Fig. 7). In the Eastern province and along the north-western border, there is a strong geographical clustering of areas transitioning from increasing trends to declining trends (dots) adjacent to substantial areas with increasing trends.

**Fig. 8:**
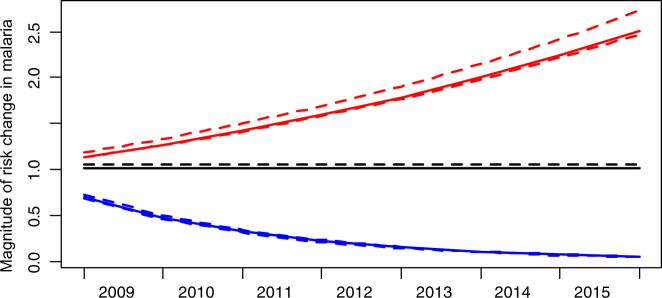
Malaria risk ratio magnitude of trend-change over time in health facilities that experienced a declining, no change or increasing trends. Colours indicate different risk ratio temporal trends: blue lines—declining linear trends; red lines—increasing linear trends, black lines—constant/ no significant change in trend; dashed lines—credible intervals at 95%, and the y-axis shows the risk ratio.

Figure 8 shows both the pooled magnitude and rate of change by trend category of those health facilities that experience a declining trend and those with an increasing trend. While it is observable that areas with declining malaria had relatively lower and rapidly declining RR, averaging from 75% as of 2009 (as the most notable decline precedes 2009) to about 25% (~50% reduced risk) by 2011. During the same period, areas with increasing malaria have a relatively higher magnitude of RR increase (60%, from 15 to 75%). Between 2011 and 2015, however, a further decline of only 25% in RR can be observed, while areas of increasing trends experienced a 2.5 times magnitude of increase in RR. However, the magnitude of RR and transmission is only measured by the trend across regions and not measured or examined for individual facilities independent of their regional trend. Further, the models do not account for any seasonal or monthly signal; therefore, the study does not show or consider their effects on observed patterns or trend variations.

#### Comparison of health facility and district-level malaria trends

A comparison of health facility results with those at the district-level^29^ *(*Supplementary Fig. 5) examined the influence of spatial scale on malaria trends to evaluate the potential and relative value as an optimal operational scale for malaria control and elimination efforts. Supplementary Fig. 6 shows the mean spatial variation of malaria trends at the health facility level. If we consider the increasing trend = 3, no change = 2, and decreasing trend = 1, we observe that 18% (13/72) of the districts have at least one health facility catchment with a higher trend in malaria, e.g. increase instead of no change or no change instead of decline. Similarly, 37.5% (27/72) of districts have at least one health facility with a malaria trend lower than the parent district, e.g. decline instead of no change or no change instead of increase. Only a few districts have a substantial mix of health facility-level trends different to the overall district trend (cross-scale), or specific differences between the two absolute trends (decrease as opposed to increasing, or vice versa) (see Supplementary Fig. 7).

Table 1 shows that 67% of health facilities exhibit the same trend as that observed in the district in which they are located. Just over 15% of health facilities have an increasing trend where the associated district has no change. In comparison, only 1.4% (24/1743) of facilities have a trend difference from a decline to an increase or vice versa. There is a statistically significant (*p* < 0.001) positive correlation (Kendall Tau_b = 0.66) between District-level trends and Health facility trends. The range of coefficients of association across several tests is from 0.51 (Cohen’s kappa) to 0.86 (Goodman and Kruskal Gamma) (Supplementary Table 4). The Bartlett test comparing variances shows an observed *χ*^2^ = 1.29, critical value = 3.84, and a computed *p* value = 0.26, showing no significant difference. This further indicates inadequate evidence to suggest that the variances between the two trends are significantly different.

**Table 1:** Trend variation between district and health facility-level trend models.

Facility trend	District trend	# of facilities	%	Total change
Decline	Increase	10	0.6%	1.4%
Increase	Decline	14	0.8%	
Increase	Increase	524	30.1%	67.5%
No change	No change	276	15.8%	
Decline	Decline	377	21.6%	
Decline	No change	58	3.3%	31.1%
No change	Decline	82	4.7%	
Increase	No change	272	15.6%	
No change	Increase	130	7.5%	

The table shows that there is little cross-trend variation in malaria risk between the district and health facility levels, with at least 67% of health facilities exhibiting the same trend observed in the district in which it is located.

#### Comparison of district and health facility-level malaria trends by population

Using Zambia’s 2015 district-level population estimates^34^, 37% (5.8 million) of people live in districts with declining malaria, 34% (5.2 million) live in districts with no trend change, and 29% (4.5 million) live in districts that have an increasing malaria trend.

At the heath facility level, the recorded total population is 13.73 million compared to the district-level total of 15.46 million, before applying a raking ratio estimation. After raking, we find similar proportions and populations living in areas with a declining trend, 37% (5.72 million) but differences in the proportions of the population with no change and those with an increasing trend, 28% (4.33 million) and 35% (5.41 million), respectively. The initial total recorded health facility-level population is 10% lower than the estimated district-level total, which could imply that the reported malaria burden and risk may not fully capture the spatio-temporal changes in treatment-seeking during the study period. For example, we may be underestimating the true community malaria burden in some areas because of the lack of population adjustments for treatment-seeking rates or overestimating due to the exclusion of the health facilities with incomplete malaria data and/or those without reliable denominators. Nonetheless, this does not affect or change the malaria trends for health facilities and districts reported or discussed, as we use a consistent method of establishing the denominator populations throughout the study period.

## Discussion

We assessed the utilisation of routine malaria surveillance data reported from health facilities to help guide local operational decision-making for intervention implementation. Spatio-temporal patterns are assessed using simplified methodologies that local malaria officers can replicate with limited modelling skills.

Nearly 70% of all health facility trends presented the same trend pattern as those of their parent district. This initially suggests that pursuing intensive malaria control or elimination efforts at the sub-district (i.e. health facility) level may not yield additional benefits vis-à-vis the additional increase in logistical and operational costs. However, health facility-level trends (Fig. 7) reveal highly relevant patterns for monitoring and planning intervention strategies going forward. We suggest that those health facility areas (>30%) where trends may be substantially different (especially higher) than the average trend of their district would be more suitable for targeted interventions. Strategies such as focal Mass Drug Administration (MDA) or reactive test and treat community strategies^35^ could help eliminate the residual foci, especially in pre-elimination and elimination areas.

The observed trends in the country’s eastern region show a very distinct geographical clustering of areas that are transitioning from increasing rates to decreasing rates. These areas are contiguous with other areas that are continually increasing, and they are predominantly along the eastern border region of the country. This is significant as this region has received intensive interventions from the government and other malaria partners as part of a targeted intervention strategy since 2013. Here we see the first evidence that a recent sub-district targeted strategy and programme implemented in the eastern region may have delivered positive, tangible outcomes even in a relatively short period of time. Nonetheless, they also highlight the importance of forging cross-border partnerships with neighbours like Mozambique to maintain this decreasing trend.

The patterns found in the southern part of the country showed a significant cluster of areas with relatively lower incidence rates transitioning from a decreasing to an increasing trend. Similar to the eastern region, these areas are contiguous with areas that have a contrasting continuous declining trend. The results show a substantial reduction in the number of areas where malaria is declining, and there may well be a deterioration in the general malaria situation that is not evident at the district level but is very obvious at the health facility level. These areas require enhanced surveillance through continuous monitoring of trends at the facility level, alongside behavioural change campaigns to encourage the population not to relax its use of malaria interventions such as nets. As the deterioration of partial immunity to malaria declines due to low exposure to the malaria parasite, these areas become more prone to outbreaks and could easily return to their former levels if care is not taken.

Our results imply that some areas of decline, as measured at the sub-district level, are experiencing a change in trend but not receiving adequate attention or appropriate levels of intervention. These areas of persistent increasing trends should be the focus of qualitative studies to understand why current interventions are not working. Further assessment of the efficacy of current tools could equally help identify where the problems lie. Our findings would also suggest that, in support of the current National Malaria Elimination Programme’s intervention strategy, a sub-district health facility-level operational programme is urgently required to stop the changing trend from decreasing to increasing rates in the southern region. The point being made here is important, especially in the context that no major national malaria policy changes were implemented during the study period, other than the consolidation of intervention scale-ups to strengthen case management, enhanced malaria testing, improved IPTp supplies, and a ramp-up of bed net distribution and IRS coverage^29^.

Given that the limitations of current tools available to health authorities are quite possibly compounding the problem, a first step would be the creation of a near real-time health facility level comparative and predictive toolset, using extant data sources, that can provide scalable spatio-temporal analyses of trends in malaria risk and rates with the capability to evaluate the impacts of localised risk factors and intervention programmes.

It is argued that as countries move closer toward elimination, more symptomatic cases may be expected to seek care, and a temporary shift in the age distribution profile is the first sign that transmission is declining^36^. Elsewhere, the authors report the opposite, finding a consistent declining trend in children <5 years but absolute increases among those aged ≥5 years^37^. Furthermore, despite the reported increases in community health worker (CHW) testing activities alongside the administration of malaria medication^29^, where we would have expected to see an increase in malaria rates, we, in fact, found significant declines in rates in both rural and urban areas. Hence, those areas where we observed actual increases in rates clearly signify a trend worth considering to prevent possible spill-overs to neighbouring areas that currently exhibit declining trends.

Our study has shown that malaria trend patterns vary depending on the measured spatial scale. Adopting a single-scale level approach (e.g. administrative districts) to monitor and implement intervention strategies may not be as efficient as a multi-scale level approach (e.g. using district and health facility service catchment areas). Our analysis suggests that, in general, district-level strategies may well be appropriate for those areas where incidence rates are high and trends are increasing. However, health facility catchment area strategies may be more efficient and effective in areas with low rates and stable or decreasing trends.

These findings are consistent with many recent findings from other studies that underscore the importance of scale and transmission setting^5,10,38,39^. For example, Bousema et al.^5^ support the contention that areas with widespread malaria transition would benefit from high-level untargeted community-wide approaches^5,40^. On the other hand, micro-scale targeting of interventions at the sub-facility level would be most effective at the “nucleated” household level, as any benefits from spill-over into the surrounding local community are limited. This approach is only suitable for areas with isolated stable hotspots and low rates but exhibiting a rising trend. Here, focused interventions like MDAs could reduce transmission without creating full-scale community-wide drug pressure. High-resolution strategies of spatially mapping vector population distributions, ecological suitability, breeding, and insecticide resistance could provide a better insight into vector-based solutions. Additionally, evaluation of new tools, drugs, vaccine pilots, and behaviour change trials will be necessary to derive new information and understanding of the complex interactions of malaria and interventions ongoing in these areas.

While approaches for spatially targeting malaria are attractive and effective in capturing residual foci of transmission and optimising the sensitivity of surveillance^41^, there is often a strong urge to target interventions at more granular levels to fast-track malaria elimination in low-transmission settings^4^. However, evidence supporting the concept of micro-hotspot intervention targeting, especially when undertaken at the individual or household levels, shows mixed results,^10^. Among other things, such as population movement, inconsistencies in observed impacts may be due to time delays between ‘hotspot’ identification and intervention deployment. This, in turn, may affect the size (geographical scope) and possibly the timing of deployments, not to mention potential problems with follow-up evaluations of hotspot stability and intervention efficiency^5,10,42^. A critical review by Stresman et al.^10^ also cautions against blind targeting of interventions simply based on the availability of granular data and encourages a combination with other additional transmission dynamics^43^.

Recent evidence shows that some contemporary spatial techniques and malaria metrics used to define micro-hotspots regularly miss important high-burden households^10^. This may partially explain why observed impacts are often not sustained or remain limited and is supported by our Supplementary Fig. 3, which highlights statistically significant cold spot clusters within broader regions of high malaria. This is also observed in the southern region among areas with low malaria, where cold spot clusters indicate a relatively lower malaria risk than the surrounding areas. Therefore, we propose that the health facility level is the optimal operationally unit for fine-scale interventions in these areas. This is because, even where reactive case detection may not be suitable (e.g. in unstable hotspots with adequate access to care^44^), health facility-level targeted interventions should still be cost-effective. Such an approach could accommodate non-endemic, hypoendemic, pre-elimination, and small-scale hotspot foci with enough coverage for a spectrum of areas ranging from large villages down to the household level for both stable and unstable scenarios (as shown in Fig. 5).

This could also have considerable implications for resource efficiency and savings through adaptive scaling and targeting interventions where they are most needed. For example, priority must be given to areas with the highest rates and increasing trends, particularly districts in Northern, Luapula and Muchinga provinces, followed by those where substantial gains have already been made, and rates are low, but they are now in a negative transition phase and at risk (i.e. Eastern, Central and parts of Lusaka provinces). This is because maintaining recent gains against malaria should be equally as important in countries like Zambia, where the primary goal is to extend those regions currently designated for malaria elimination. Doing so, however, requires consistency in both strategy and the application of interventions.

Our study also suggests that the relative increase in Zambia’s malaria incidence is mostly characterised by a north-south transmission pattern and one that is closely associated with proximity to the borders of higher incidence neighbouring countries such as the Democratic Republic of Congo (DRC) and Mozambique, both of which are in the top five highest malaria incidence countries globally^45–50^ during 2011–2019.

These observations demonstrate the importance for countries to actively pursue collaborative cross-border malaria initiatives with highly endemic neighbouring countries. For Zambia, this is essential as parts of the country progress towards eliminating malaria. Strong cross-border collaborations exist along Zambia’s southern border, funded through regional initiatives of the Elimination 8 (E8)^51,52^, but evidence of similar initiatives with the DRC has yet to be seen. Functioning bilateral operational initiatives must be pursued if Zambia must make significant headway with its control efforts in these border regions. As the E8 states, 'a country will never achieve or sustain malaria elimination as long as transmission continues in neighbouring countries^53^' because mosquitoes do not respect political boundaries.

With the ever-improving quality and increasing availability of routinely collected malaria data, health facility level reporting provides a unique opportunity for Zambia and other endemic malaria countries to gain greater insights into pre/elimination transmission dynamics and offers alternative operationalisation strata for malaria programmes at the health facility level. We encourage countries to take advantage of health facility-level data granularity to test new strategies that previously were deemed operationally unfeasible. Nonetheless, this does not preclude the need for adaptive scaling, especially where the transmission scenario is favourable and supports residual foci investigations. Household-level investigations could collect relevant additional information on the nature of infections, case transmission dynamics (such as imported vs local, relapsing vs recrudescent), parasite species monitoring and serology studies.

Potential limitations of this study include those relating to the population denominators used to standardise malaria counts and calculate incidence rates. Although the population used here for calculating malaria incidence rates at the health facility level is official estimates from district census figures, they have accuracy issues. A combination of health facility population headcounts and official census estimates are the primary sources of population data. However, the health facility-level catchment areas are not part of the regular census or officially recognised population count administrative units for data collection. Thus, there is an inherent potential limitation with the accuracy of denominator service populations, which may be imprecise, and may influence some of the observed facility-level variations in incidence rates, causing under or overestimations^54^.

Nonetheless, the population denominators used in this study represent the best available official dataset currently used by the Ministry of Health for their day-to-day decision-making, and the results obtained here remain comparable to those generated by the programme. It remains challenging to accurately ascertain the true catchment areas of the population attending specific health facilities, as many geographic and other treatment-seeking behaviours influence the choice of health facility^55,56^.

We acknowledge that, while not the focus of this paper, adding covariates that are strongly associated with malaria transmission, such as environmental and socio-demographic factors^37,57–59^, including intervention scale-up, would have enabled us to quantify their influence on overall malaria trends in many parts of the country^37,60–62^. Based on previous studies^57,58^, climatic variability and change showed a strong association with increasing trends, while high intervention coverage of IRS and ITNs was associated with declining malaria in Zambia. Although we believe that the effects of covariates are already accounted for in the data and, along with any confounding effects, should have been captured by the non-spatial random effects component in the model, uncertainty on the estimates of this influence from covariates remains. Additionally, the available scale level of most relevant covariates, such as rainfall and interventions, would have rendered them unusable^63^ in this fine-scale analysis without substantial additional preparatory work or moving to pixel-level analysis. Nonetheless, it remains an opportunity for further study to understand the interactional effects of interventions with the physical and social-environmental variables and how these implicitly influence the observed trends. This is the focus of a follow-up paper.

## Conclusion

Our study has demonstrated the value of contemporaneously establishing national-level monitoring and reporting of malaria incidence and trends at both the district and health facility levels. We have shown that the health facility level could provide the most relevant and operationally viable option for capturing key malaria dynamics, such as the underlying spatio-temporal incidence rates, risk trends, and hotspot clusters that support an adaptive scaling approach to intervention planning. We would suggest that countries re-assess and carefully reconsider their malaria programmes and strategies in this regard. Using the approach and methods presented here, governments can quickly identify the most appropriate scale levels (i.e. district, facility, or household) for more efficient planning and operationalisation of intervention strategies.

The importance of border effects on rates, trends, and observed malaria burden^64^ for malaria elimination strategies in countries like Zambia cannot be overemphasised. Our nationwide health facility-level analysis in Zambia identified significant clusters of sub-district-level variations in malaria trends that are negative in some regions in the northeast, south-central, and southwest but positive in northern regions, typically concentrated along border areas with Mozambique and the DRC. The clear message is that pursuing malaria elimination alongside neighbours with a poorer malaria epidemiological status calls for the urgent formation of meaningful bilateral cross-border malaria initiatives. In the case of Zambia, the DRC, Angola and Mozambique border areas must be an immediate priority.

## Supplementary information

Supplementary Information

Reporting Summary

## Data Availability

The data used is considered the property of the Republic of Zambia and, therefore, subject to ethical and legal restrictions. This means that although it can freely be requested through the Ministry of Health, it cannot be shared without prior approval from the Ministry. Permission to use or access the district-level malaria datasets for mortality and morbidity used in the manuscript can be obtained from the National Malaria Elimination Centre (NMEC) through *The Ministry of Health, Ndeke House, Haile Selassie Avenue, P.O Box, 30205, Lusaka (Zambia); Email: ps@moh.gov.zm*.
