# Supplementary material for: Spatio-temporal monitoring of health facility-level malaria trends in Zambia and adaptive scaling for operational intervention
Source: Commun Med (Lond). 2022 Jul 1;2:79. doi: 10.1038/s43856-022-00144-1 (PMC9249860; doi:10.1038/s43856-022-00144-1)
Supplement: Supplementary file 1 — Supplementary Information [file 43856_2022_144_MOESM1_ESM.pdf]

Supplementary Methods and Results

Spatio-temporal monitoring of health facility-level malaria trends in Zambia and adaptive scaling for operational intervention

Jailos Lubinda*[1,2,3,4], Yaxin Bi[5], Ubydul Haque[6,7,8], Mukuma Lubinda[9], Busiku Hamainza[10], Adrian J. Moore[1]

Health facility spatio-temporal models

During the period of this study from 2009 to 2015, the number of health facilities reporting in the health management information system (HMIS) increased from 1552 in 2009 to about 1869 in 2015. The increase was made up of a combination of government, mission (faith-based), and private providers [1–4]. During the same period, Zambia's population increased by 18.5%, while the total number of health facilities increased by 18.7%, and the ratio of health facilities to population ratio remained the same throughout (1.2 health facilities per 10 000 population). Based on a comparison between population growth and the increase in health facilities, it is proposed that the construction of new health facilities was primarily driven by population growth and, as such, can be argued that given the population growth ratio was consistent with the provision of health care facilities per person, it could logically be assumed that both incidence rates and trends of malaria should have remained constant unless other factors were at play. Such factors could include – climate variability (favouring malaria infections), the geographical variance of sub-district malaria intervention distribution, or associated differences in cultural attitudes towards the utilisation of malaria interventions, all of which could influence the effectiveness of interventions against malaria.

In addition, 76 health facilities were excluded due to incomplete malaria data, the lack of both malaria and population data, or more specifically, the absence of a reliable population denominator. Malaria cases reported at these 76 health facilities accounted for 0.8% of the total reported malaria between 2009 and 2015 available for analysis. No imputation methods were applied because information on missingness was not available.

The dataset was divided into two sets, with a separation between 1743 low-level health facilities from 100 higher-level hospitals. We grouped health posts, health centres, and clinics, together with all private health facilities into the first group for analysis of lower-level (primary care) health facilities where most of the malaria outpatient department (OPD) screening and treatment happens. The other set comprised only public level 3 and referral (secondary/tertiary care) hospitals where malaria cases treated are the most severe and often involve admissions. Given that most of the severe malaria cases received at hospitals are referrals from primary health facilities within hospital catchments or the Hospital Affiliated Health Centres (HAHC)[5]. HAHCs act as onsite OPDs for the hospitals, which meant that including referral hospitals would more likely lead to double counting of cases already received at the health centre level before the referral rather than missing cases out. This separation helped us avoid double-counting malaria cases referred to hospitals from lower-level health facilities. All private hospitals were included in the first group because they also provide services offered by lower-level public health facilities.

The implication of the changing number of health facilities

Due to the periodic construction of new health facilities and upgrading of other lower facilities to higher-level functions, the number of health facilities with at least one case reported in the

HMIS increased by about 600 between 2012 and 2016. While this increase in newly constructed health facilities captured in the reporting system may be interpreted as a possible explanation for why there may be increases in malaria cases, based on the point made above regarding the virtually identical corresponding increase in population, we would argue that in fact, this has little to do with observed increases in malaria, and instead is simply a reflection of enhanced access, and possible improvements to the quality of health services provided. This is particularly true for urban areas where more people tend to seek health facility services relative to the planned/expected facility capacity or in rural areas where some villages are far from the available rural health facilities.

Nonetheless, most new centres still take a long time to gain the trust of people around them as most patients would still opt for the facility they have previous experience with even if this is further away [6]. Such is often observed in people's frequent preference for hospitals over health facilities even though the health centre nearer to their location is adequately equipped to handle the same disease. We would propose that while the changing number of health facilities improves the quality of health services offered, it does not affect the actual long-term district or health facility trend reporting but it may temporarily affect time point-specific records at surrounding health facilities.

Supplementary Results

<u>Low Spatial (intra-district) district–level and temporal (annual) trend variation</u>

The mean posterior exceedance probabilities of health facilities in more northern areas are higher (Supplementary Figure 6) than those in the southern region. The same is true with posterior median estimates (Supplementary Figure 7) which show a similar spatial transitional pattern generally higher in the north compared to the southern areas. The overall model in INLA, with type II interaction, was the best model compared to Type I, III, and IV. It had a marginal log-Likelihood of -103348.76, and the linear predictor and fitted posterior marginal decomposition of the total amount of variability from log rates was 68.05% 3.32%, and 28.63% for Spatial, Temporal, and Space-time, respectively. This model confirms little temporal variance, while the bulk of the variance could be explained by the spatial and the spatio-temporal components. The details of the posterior marginal distributions using precision and variance scales are given in Supplementary Tables 3 and Supplementary 4.

REFERENCES

1. Zambian Ministry of Health. The 2012 List of Health Facilities in Zambia: Preliminary Report, v15. Ministry of Health Lusaka; 2013.

2. CSO. 2010 Census of population and housing: National Analytical Report. Natl Anal Rep. 2012;

3. Ferrinho P, Siziya S, Goma F, Dussault G. The human resource for health situation in Zambia: Deficit and maldistribution. Hum Resour Health. 2011;

4. Hoppenbrouwer J, Kanyengo CW. Current access to health information in Zambia: A survey of selected health institutions. Health Info Libr J. 2007;

5. Comfort AB, van Dijk JH, Mharakurwa S, Stillman K, Gabert R, Korde S, et al. Hospitalizations and costs incurred at the facility level after scale-up of malaria control: pre-post comparisons from two hospitals in Zambia. Am J Trop Med Hyg [Internet]. 2013/11/11. The American Society of Tropical Medicine and Hygiene; 2014;90:20–32. Available from: https://pubmed.ncbi.nlm.nih.gov/24218409

6. Nguyen M, Howes RE, Lucas TCD, Battle KE, Cameron E, Gibson HS, et al. Mapping malaria seasonality in Madagascar using health facility data. BMC Med. BioMed Central; 2020;18:1–11.

Supplementary Tables and Figures

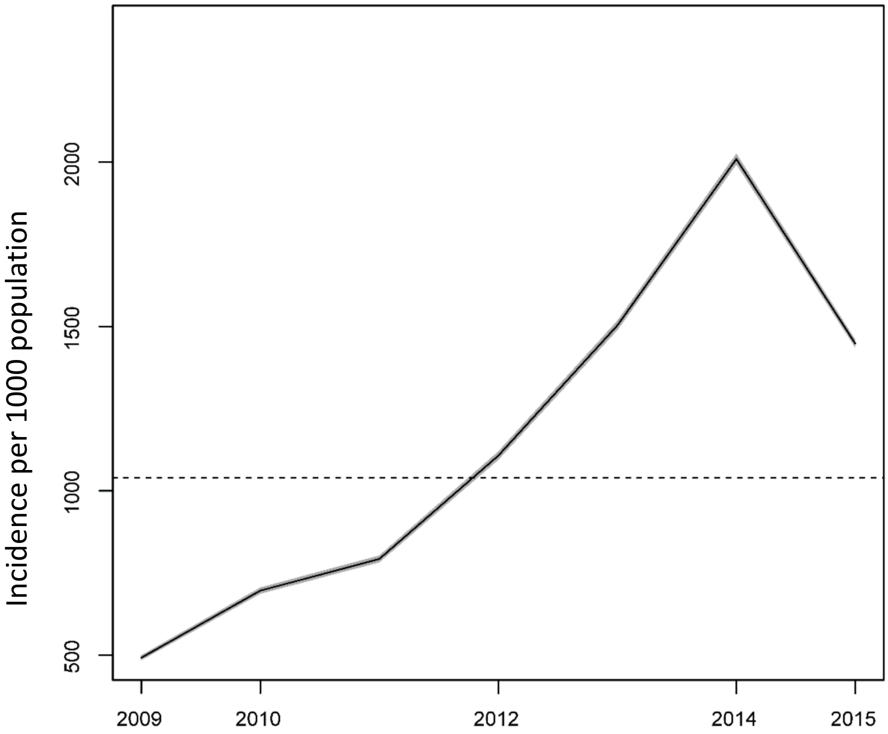

Supplementary Figure 1: Mean temporal incidence rates

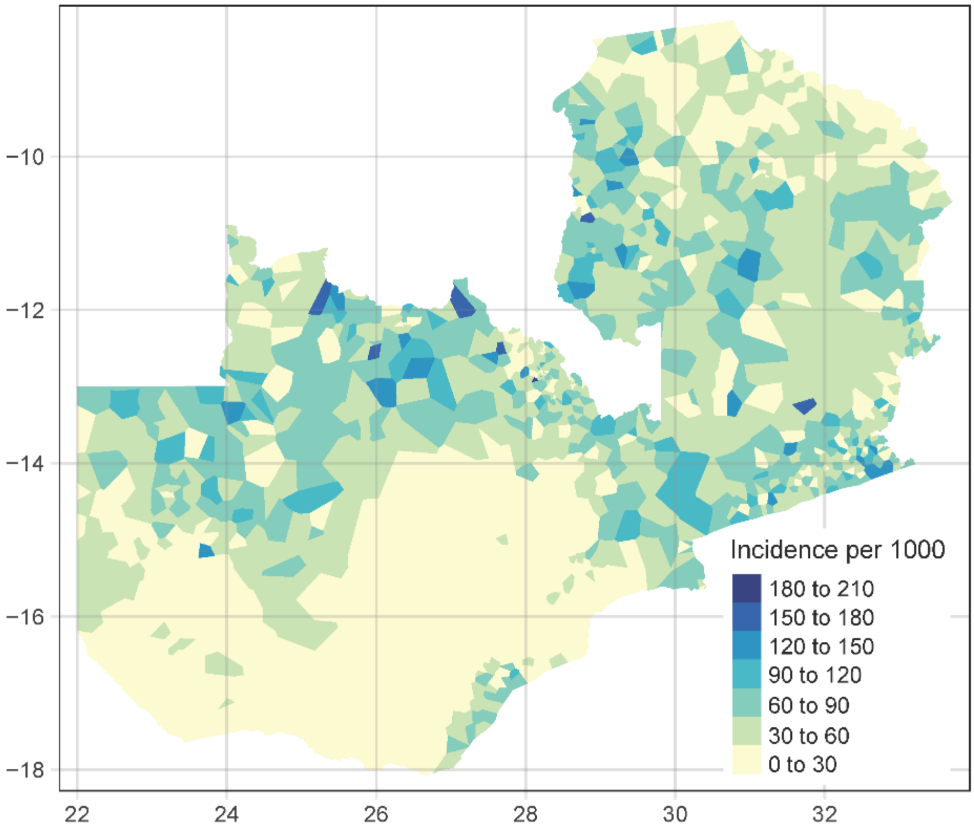

Supplementary Figure 2: Posterior median estimates of malaria incidence rates per 1000, 2009 – 2015. The figure shows the pattern of the mean geographical distribution of malaria incidence at the health facility level.

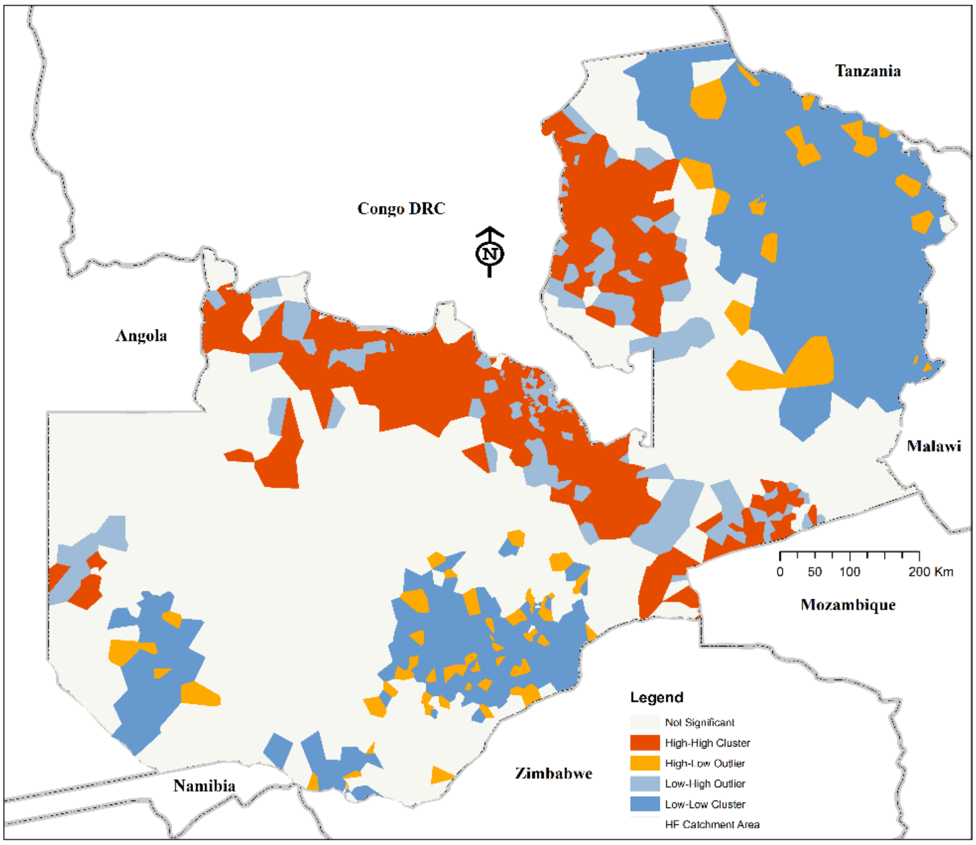

Supplementary Figure 3: Outliers within both hotspots and cold spot areas

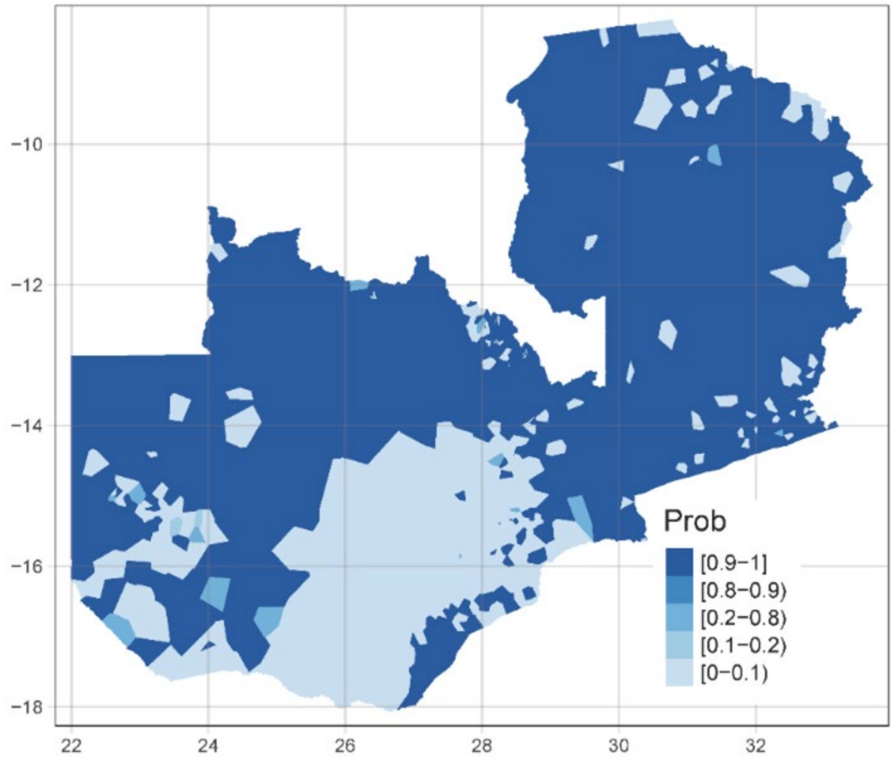

Supplementary Figure 4: Mean malaria incidence posterior exceedance probabilities

The scale and legends show a probability scale from 0 – 1, where 0.9 - 1 denotes the highest probability of getting malaria and 0 - 0.1 denotes the lowest.

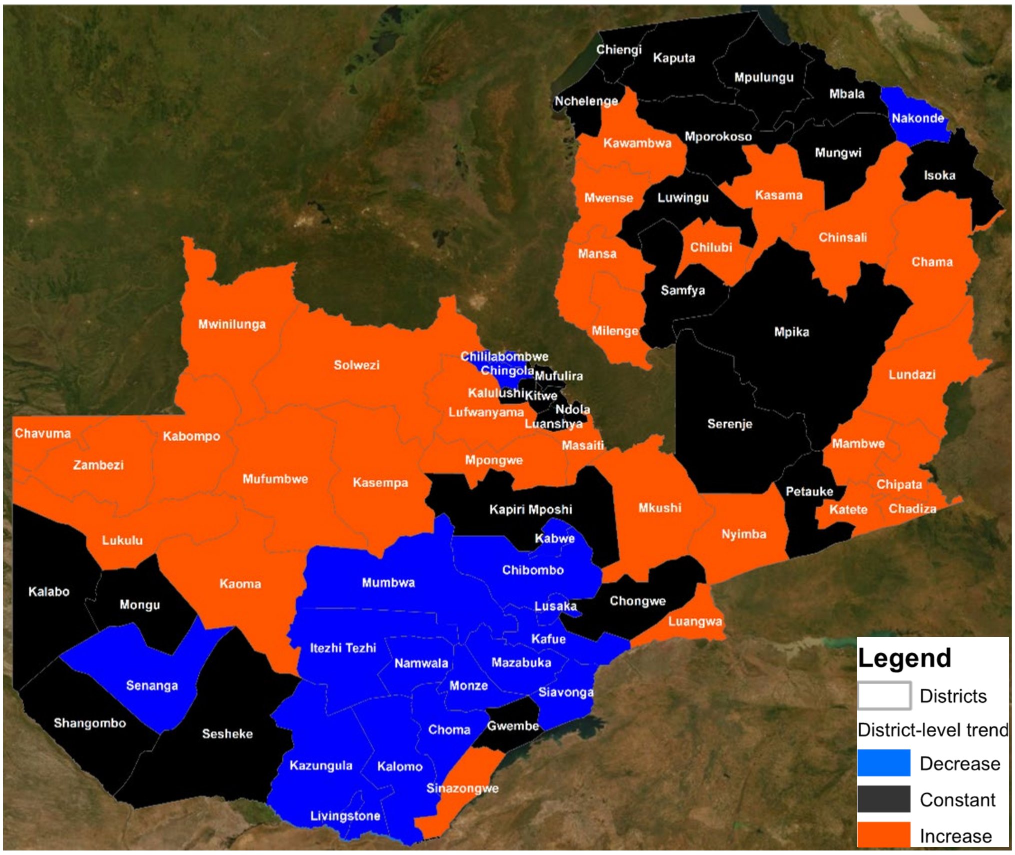

Supplementary Figure 5: District-level malaria trends between 2000 and 2015

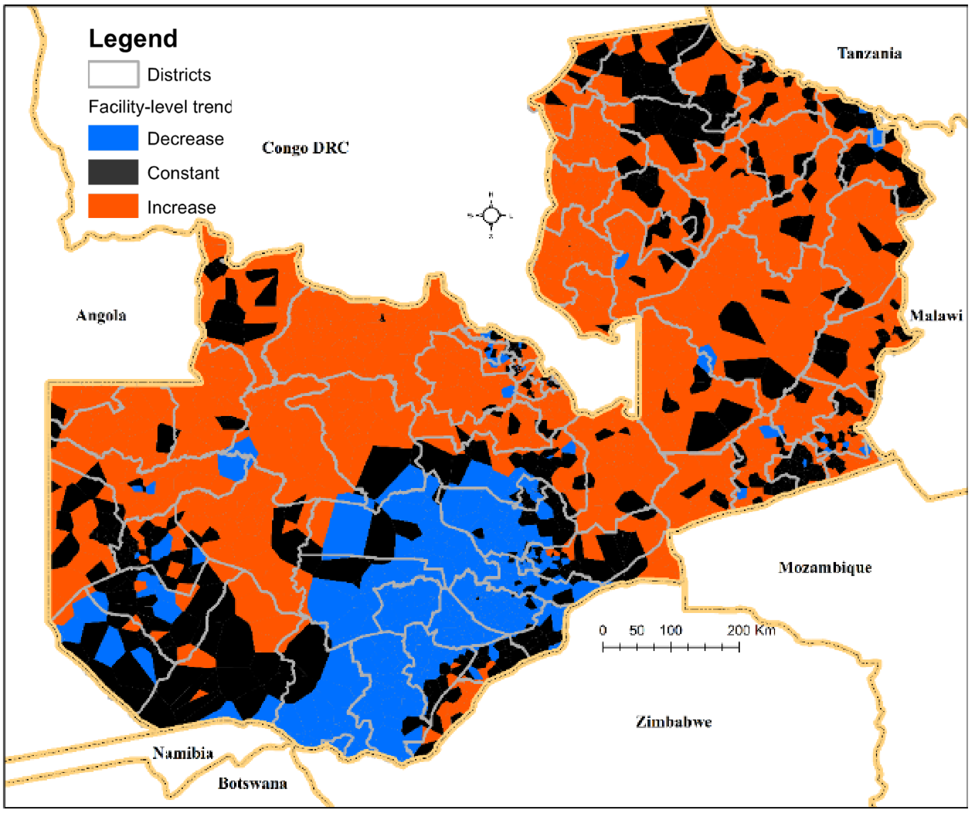

Supplementary Figure 6: Health Facility malaria trends

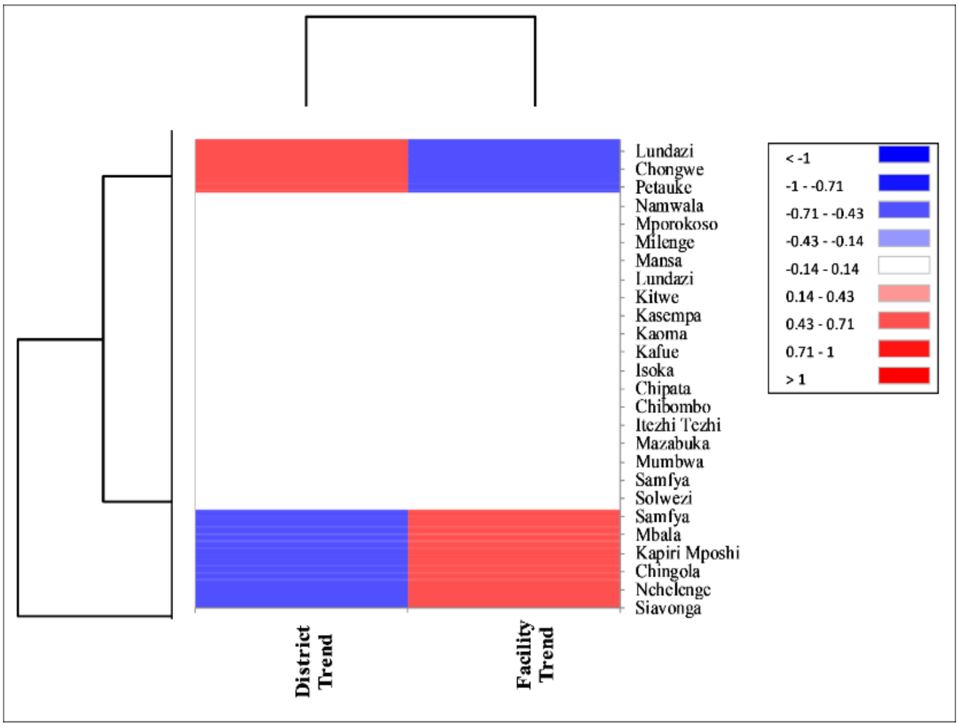

Supplementary Figure 7: Heat map of trend differences significance between district and HF models

The Fig. shows the districts that had significant differences in trends between district vs health facility level models. It shows which districts what proportion of health facilities with a different trend compared to the trend in the district model; whether decreasing (*blue*) vs increasing (*red*) malaria trend models at HF vs district trends

	Mode	2.5%	97.5%	n.sample	%accept	n.effective	Geweke.diag
(Intercept)	0.0104	0.0098	0.0486	10000	43.7	193.7	-2.0
gamma.LD	-0.3770	-0.3870	-0.3765	10000	40.5	136.3	1.7
gamma.LI	0.1139	0.1120	0.1194	10000	41.4	3721.9	-4.7
lambda.Constant	0.2575	0.2359	0.3739	10000	100.0	243.8	-2.3
lambda.LD	0.2668	0.2440	0.2876	10000	100.0	2552.4	5.3
lambda.LI	0.4725	0.3617	0.4956	10000	100.0	288.1	2.4
tau2	0.2172	0.1648	0.2583	10000	100.0	434.7	4.8
rho	0.2778	0.1759	0.3667	10000	44.0	1175.6	4.6

Supplementary Table 1: A summary of the estimated trends of malaria risk
The table also shows estimates within 95% credible intervals of significance. The tau2 and rho are estimators of variance parameters and spatio-temporal dependence or autocorrelation in the model, and all results here are significant statistically significant. The Table trend results are expressed as lambda.LD for [(λ) Linear decline (LD)], lambda.Constant, and lambda.LI for [(λ) Linear Increase (LI)], respectively.

	mean	sd	2.5%	50%	97.5%
Intercept	-4.6	0.061	-4.7	-4.6	-4.5
Spatial variance component	3.1	0.11	2.9	3.1	3.4
Spatial smoothing parameter	0.37	0.029	0.32	0.36	0.43
Temporal variance component	0.11	0.075	0.023	0.088	0.3
Temporal (non-structured) variance component	0.21	0.089	0.0058	0.23	0.28
Spatio-temporal variance component	2.1	0.021	2	2.1	2.1

Supplementary Table 2: Summary statistics for the variance Scale (mean, standard deviation and quantiles)

	mean	sd	2.5%	50%	97.5%
Intercept	-4.6	0.061	-4.7	-4.6	-4.5
Spatial precision parameter	0.32	0.011	0.3	0.32	0.34
Spatial smoothing parameter	0.37	0.029	0.32	0.36	0.43
Temporal precision parameter	14	11	3.3	11	44
Temporal (non-structured) precision parameter	5.4e+02	4.3e+03	3	76	3.6e+03
Spatio-temporal precision parameter	0.48	0.005	0.47	0.48	0.49

Supplementary Table 3: Summary statistics for the precision Scale (mean, standard deviation and quantiles)

Correlation Test	Coefficient	LCI 95%	UCI 95%	Correlation Test	Coefficient
Goodman and Kruskal Gamma	0.859	0.835	0.883	Pearson's Phi	0.83
Kendall's tau	0.656	0.629	0.683	Contingency coefficient	0.639
Stuart's tau	0.064	0.611	0.669	Cramer's V	0.587
Somers D (R/C)	0.646	0.619	0.673	Tschuprow's T	0.587
Somers D (C/R)	0.666	0.638	0.694	Cohen's kappa	0.505

Supplementary Table 4: High correlation between District and Health facility trends
