# Supplementary material for: Spatio-temporal monitoring of health facility-level malaria trends in Zambia and adaptive scaling for operational intervention
Source: Commun Med (Lond). 2022 Jul 1;2:79. doi: 10.1038/s43856-022-00144-1 (PMC9249860; doi:10.1038/s43856-022-00144-1)
Supplement: Supplementary file 2 — Reporting Summary [file 43856_2022_144_MOESM2_ESM.pdf]

nature portfolio

Corresponding author(s): Jailos Lubinda

Last updated by author(s): Jun 12, 2022

Reporting Summary

Nature Portfolio wishes to improve the reproducibility of the work that we publish. This form provides structure for consistency and transparency in reporting. For further information on Nature Portfolio policies, see our Editorial Policies and the Editorial Policy Checklist.

Statistics

For all statistical analyses, confirm that the following items are present in the figure legend, table legend, main text, or Methods section.

n/a	Confirmed	
☐	☒	The exact sample size (n) for each experimental group/condition, given as a discrete number and unit of measurement
☒	☐	A statement on whether measurements were taken from distinct samples or whether the same sample was measured repeatedly
☐	☒	The statistical test(s) used AND whether they are one- or two-sided<br>*Only common tests should be described solely by name; describe more complex techniques in the Methods section.*
☒	☐	A description of all covariates tested
☐	☒	A description of any assumptions or corrections, such as tests of normality and adjustment for multiple comparisons
☐	☒	A full description of the statistical parameters including central tendency (e.g. means) or other basic estimates (e.g. regression coefficient) AND variation (e.g. standard deviation) or associated estimates of uncertainty (e.g. confidence intervals)
☐	☒	For null hypothesis testing, the test statistic (e.g. F, t, r) with confidence intervals, effect sizes, degrees of freedom and P value noted<br>*Give P values as exact values whenever suitable.*
☐	☒	For Bayesian analysis, information on the choice of priors and Markov chain Monte Carlo settings
☐	☒	For hierarchical and complex designs, identification of the appropriate level for tests and full reporting of outcomes
☐	☒	Estimates of effect sizes (e.g. Cohen's d, Pearson's r), indicating how they were calculated

Our web collection on statistics for biologists contains articles on many of the points above.

Software and code

Policy information about availability of computer code

Data collection	No Software was used.
Data analysis	We used R programme (v. 4.0) to run the models and determine trend clusters. We also used ArcGIS v10.6 for the creation of some maps.

For manuscripts utilizing custom algorithms or software that are central to the research but not yet described in published literature, software must be made available to editors and reviewers. We strongly encourage code deposition in a community repository (e.g. GitHub). See the Nature Portfolio guidelines for submitting code & software for further information.

Data

Policy information about availability of data

All manuscripts must include a data availability statement. This statement should provide the following information, where applicable:

- Accession codes, unique identifiers, or web links for publicly available datasets
- A description of any restrictions on data availability
- For clinical datasets or third party data, please ensure that the statement adheres to our policy

All data associated with this study are present in the paper or the Supplementary Materials. No custom made code was used in the study, however, code to reproduce study findings would be made available by request from the corresponding author.

Field-specific reporting

Please select the one below that is the best fit for your research. If you are not sure, read the appropriate sections before making your selection.

☒ Life sciences ☐ Behavioural & social sciences ☐ Ecological, evolutionary & environmental sciences

For a reference copy of the document with all sections, see nature.com/documents/nr-reporting-summary-flat.pdf

Life sciences study design

All studies must disclose on these points even when the disclosure is negative.

Sample size	As this is a retrospective study, the sample was 146 000 observations
Data exclusions	Data from 100 level 3 secondary/tertiary care public hospitals was excluded from the study, which according to policy where malaria cases treated at these facilities are comprised of severe and often referral involve admissions, which if included would have resulted in double counting of cases as such hospitals would count referrals that have already been counted at the initial screening health facility. Besides, these hospitals often have a larger service catchment/population that includes several health facilities under them, and their populations overlap with health facility level denominator populations.
Replication	To verify reproducibility, early models were run in R independently before reproducing them using the shiny SSTCDapp. The huge datasets were also run in the shiny SSTCDapp to leverage the processing speed and memory.
Randomization	Not Applicable to this study, as this is a analysis was done on all samples collected. Nonetheless, Randomized Dependence Coefficient and other methods were used to analyze the influence of independent factors on the dependent variables.
Blinding	Not applicable because the data is from routine collection and sample analysis is done by independent staff with no link to investigators, and aggregated data was received.

Reporting for specific materials, systems and methods

We require information from authors about some types of materials, experimental systems and methods used in many studies. Here, indicate whether each material, system or method listed is relevant to your study. If you are not sure if a list item applies to your research, read the appropriate section before selecting a response.

Materials & experimental systems

n/a	Involved in the study	
☒	☐	Antibodies
☒	☐	Eukaryotic cell lines
☒	☐	Palaeontology and archaeology
☒	☐	Animals and other organisms
☒	☐	Human research participants
☒	☐	Clinical data
☒	☐	Dual use research of concern

Methods

n/a	Involved in the study	
☒	☐	ChIP-seq
☒	☐	Flow cytometry
☒	☐	MRI-based neuroimaging
